# Structure-Based Design of Peptides Targeting VEGF/VEGFRs

**DOI:** 10.3390/ph16060851

**Published:** 2023-06-07

**Authors:** Rossella Di Stasi, Lucia De Rosa, Luca Domenico D’Andrea

**Affiliations:** 1 Istituto di Biostrutture e Bioimmagini, CNR, 80131 Napoli, Italy; rossella.distasi@cnr.it (R.D.S.); lucia.derosa@cnr.it (L.D.R.); 2 Istituto di Scienze e Tecnologie Chimiche “G. Natta”, CNR, 20131 Milano, Italy

**Keywords:** peptide, VEGF, VEGF receptor, peptide design, antiangiogenic, protein binding epitopes, receptor binder, molecular mimicry

## Abstract

Vascular endothelial growth factor (VEGF) and its receptors (VEGFRs) play a main role in the regulation of angiogenesis and lymphangiogenesis. Furthermore, they are implicated in the onset of several diseases such as rheumatoid arthritis, degenerative eye conditions, tumor growth, ulcers and ischemia. Therefore, molecules able to target the VEGF and its receptors are of great pharmaceutical interest. Several types of molecules have been reported so far. In this review, we focus on the structure-based design of peptides mimicking VEGF/VEGFR binding epitopes. The binding interface of the complex has been dissected and the different regions challenged for peptide design. All these trials furnished a better understanding of the molecular recognition process and provide us with a wealth of molecules that could be optimized to be exploited for pharmaceutical applications.

## 1. Introduction

The development of molecules able to modulate the vascular endothelial growth factor (VEGF)-dependent angiogenic response is crucial to treat diseases depending on excessive vascularization, such as rheumatoid arthritis and psoriasis, degenerative eye conditions and tumor growth and metastasis, or on insufficient angiogenesis, such as ulcers and ischemic heart disease. Furthermore, molecules able to stimulate appropriate vascular support are also essential in tissue regenerative applications.

Several types of molecules have been proposed to modulate (mainly to inhibit) the angiogenic response: antibodies; small organic molecules, especially tyrosine kinase inhibitors (TKIs); and peptide-like molecules. This last class of molecules has been largely explored as it presents the optimal features for targeting large protein–protein interaction surfaces as in the case of molecules targeting the complex VEGF/VEGF receptors (VEGFRs).

For these reasons, the research of novel peptides for modulating the biological activity of the VEGF/VEGFR molecular system has been a fervent field in the last few years. Several approaches have been used: screening of linear protein sequences, phage-display peptide library, synthetic libraries and structure-based design. In this review, we intend to highlight the work conducted to develop molecular binders by structure-based design of peptides mimicking VEGF/VEGFR binding epitopes. The structure-based design of peptides targeting VEGF or its receptors has been mainly based on the identification of protein binding regions which can involve protein secondary structure motifs or loops, including the hot spot residues. These binding epitopes are the molds for modeling the peptide structures to obtain the binders which could be conformationally constrained to induce molecular stability and optimal tridimensional arrangements of the interacting side chains for target recognition.

## 2. The Biology of VEGF/VEGFR Molecular System

**Figure 1:**
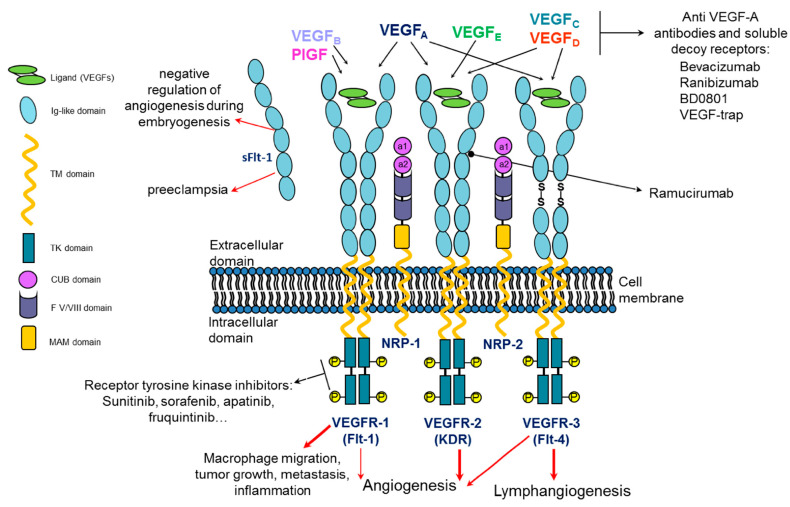
Schematic representation of VEGF/VEGF receptor molecular system and its architecture. Three full-length receptors and one soluble form of VEGFR-1 are illustrated together with the homodimeric VEGF, its homologs and their selectivity of binding to the three receptors. Upon VEGF binding, VEGFRs are activated, triggering intracellular signaling regulating physiological and/or pathological processes. NRP-1 and -2 VEGFRs co-receptors are depicted. Examples of targeting molecules against VEGF isoforms, VEGFR ectodomain and intracellular kinase domain are listed.

VEGF and its receptors play a pivotal role in the regulation of angiogenesis and lymphangiogenesis in vertebrates, driving the sprouting of new blood/lymph vessels from existing ones not only in physiological but also in pathological conditions including cancer. Angiogenesis is a process particularly active during embryogenesis, while during adult life, it is quiescent and limited to physiological phenomena such as the ovarian cycle, wound healing and tissue growth and repair [1]. In fact, endothelial cells (ECs) are considered stable and almost quiescent, showing limited turnover in the adult vasculature, even if they retain their plasticity and the ability to sense and respond to angiogenic stimuli, reactivating when it is required [2]. VEGF-A is the most studied member of the VEGF gene family. It is encoded by a single gene consisting of eight exons and seven introns which maps to chromosome 6 in humans. VEGF-A is part of a growth factor family which includes several homodimeric homologs glycoproteins such as placental growth factor (PlGF), VEGF-B, VEGF-C, VEGF-D and VEGF-E encoded by the parapoxvirus orf virus. All these structurally related glycoproteins organize into antiparallel homodimers characterized by a highly conserved structural motif known as the cystine knot motif, consisting of two disulfide bridges with a third disulfide bond crossing them [3]. The growth factors of the VEGF family elicit their biological activity through the binding to three receptor tyrosine kinases denoted as vascular endothelial growth factor receptors 1, 2 and 3 (VEGFR-1, -2 and -3) (Figure 1). These VEGFRs are typically composed of an extracellular ligand-binding portion (ECD) consisting of seven immunoglobulin (Ig)-like domains, a transmembrane domain and an intracellular tyrosine kinase domain that represents the most conserved region among the three receptors, with high sequence identities [4]. Upon binding to the extracellular portion of VEGFRs, VEGF and the other members of the growth factor family lead to the dimerization of the receptors that, consequently, undergo auto-phosphorylation of their intracellular kinase domain, triggering the activation of downstream signaling pathways (PI3K/AKT, PKC, PLCγ, Raf/Ras, MAPK/ERK) resulting in angiogenic stimuli [5]. VEGF and its homologs show different functions and exert their biological activity by selectively binding to and activating the three VEGF receptors as depicted in Figure 1 [6,7]. PlGF and VEGF-B exclusively bind to VEGFR-1, but the latter, unlike VEGFR-2, does not play a relevant role in physiological angiogenesis in adults, while it is important in tumor-associated angiogenesis [5]. An important feature of VEGFR-1 is that, unlike other VEGFR genes, it expresses two types of mRNA: one for the full-length receptor and another for a soluble short protein known as soluble VEGFR-1 (sFlt-1) consisting in the first six Ig-like domains of its extracellular portion (Figure 1). Because of the capability of this soluble form sFlt-1 to trap VEGF ligands, VEGFR-1 plays a negative role in angiogenesis during embryogenesis. In pathological settings instead, VEGFR-1, which is expressed not only on ECs but also on other cell types, such as macrophages, is able to promote inflammatory diseases, cancer metastasis and atherosclerosis upon binding of its specific ligand PlGF and activation of its kinase domain. Moreover, both VEGFR-1 and PlGF are expressed in a variety of tumors, promote invasiveness and contribute to resistance to anti-VEGF-A therapy [8]. VEGF-C and VEGF-D show the same receptor binding specificity to both VEGFR-2 and -3, but not to VEGFR-1, resulting in mitogens for vascular and lymphatic endothelial cells [9]. The VEGF-E homolog encoded by the orf virus binds and activates only and specifically VEGFR-2. VEGF-A elicits its mitogenic activity, resulting in EC migration and survival and the formation of new vessel branches, by binding to receptors VEGFR-1 and VEGFR-2 (Figure 1). Interestingly, the affinity of VEGF-A to VEGFR-1 is about 1 order of magnitude higher than that to VEGFR-2, but the tyrosine kinase activity of VEGFR-2 in response to VEGF-A is much higher (about 10-fold) than that of VEGFR-1, whose activation kinase domain has weak activity due to the lack of positive regulatory sites of phosphorylation [6,10].

All VEGF family members also interact with other types of membrane receptors: neuropilin-1 and -2 (NRP-1, NRP-2). NRPs lack cytoplasmic enzyme activity and, consequently, direct signaling capabilities and act as co-receptors of VEGFRs. Recently, NRP-1 has also been discovered as a co-receptor for SARS-CoV-2 viral entry, along with ACE2, and has thus become one of the COVID-19 research foci [11,12].

Considering the wide scenario involving the complex VEGF molecular system and the biological processes by which angiogenesis is activated, in the last few years, a growing interest has arisen in the predominant role of VEGF in processes other than the well-established physiopathological conditions such as cancer, ocular diseases and wound healing. Recently, the role of VEGF/VEGFRs in cellular rejuvenation and aging [13], as a neurotrophic factor for motoneurons [14] or as an endogenous antioxidant [15], has been highlighted.

To date, several pieces of structural information on VEGF/VEGFR family members have been reported based on NMR, X-ray crystallography, small-angle X-ray scattering and single-particle electron microscopy, elucidating how VEGF ligands bind to the Ig-like domains present in the extracellular portion of the receptors [16]. These data are essential for the development of molecules able to target the VEGF/VEGFR molecular system, modulate the VEGF-dependent angiogenesis and counteract the pathological aspects correlated to excessive and/or defective angiogenesis.

## 3. VEGF/VEGFR-Targeting Peptides: An Opportunity in Pharmaceutical Sciences

Therapeutic angiogenesis is sought as the ultimate intervention to solve chronic ischemia and cardiovascular diseases in those conditions that cannot be treated alternatively. Furthermore, advances in therapeutic angiogenesis have been realized through the use of engineered biomaterials or by delivering angiogenic molecules as recombinant proteins [17]. Its converse, antiangiogenic treatments aimed at the blockage of the VEGF/VEGFRs axis, are a promising strategy in oncology and ocular pathologies.

Currently, there are two major antiangiogenic therapeutic approaches: neutralization of VEGF/VEGFRs by monoclonal antibodies or engineered proteins that mimic VEGFRs and blockage of VEGF receptor kinase activity with small molecule inhibitors (TKIs) [5]. The first category includes U.S. Food and Drug Administration (FDA)-approved molecules such as the humanized monoclonal antibody bevacizumab (Avastin) and the humanized Fab-fragment ranibizumab (Lucentis) approved in 2004 and 2006, respectively, as anti-VEGF-A agents. The former was initially approved as the first-line treatment of metastatic colorectal cancer in combination with chemotherapy, but now its use includes several other aggressive carcinomas such as metastatic breast cancer, non-small-cell lung cancer, glioblastoma, renal cell carcinoma, ovarian cancer and cervical cancer [18]. Ranibizumab (Lucentis) is employed for the treatment of neovascular age-related macular degeneration, as is the soluble decoy receptor VEGF-Trap (Aflibercept) that shows high affinity for all VEGF-A isoforms and was approved in 2011 [19]. Ramucirumab (Cryramza), a humanized monoclonal antibody targeting the extracellular domain of the VEGFR-2, was approved in 2014 for patients with advanced/metastatic gastric or gastroesophageal junction adenocarcinoma, and the humanized rabbit anti-VEGF monoclonal antibody BD0801 is in the phase III clinical developmental stage [20,21]. Sunitinib, sorafenib, apatinib, pazopanib and fruquintinib are instead examples of multikinase inhibitors, approved by the FDA for the treatment of several severe carcinomas [22,23]. However, although neutralizing antibodies such as bevacizumab show a long bloodstream half-life and a high specificity for the target, they suffer from limitations due to the high production costs and their drawbacks in the clinic related to intravenous dosing and to immunogenicity after long-time treatments. On the other hand, TKIs, especially the earlier generation, show poor kinase specificity that translates into a less optimal duration of inhibition of any target. A valid alternative to drugs such as those described above involves peptide-based biomolecules.

In the last few decades, pharmaceutical companies invested increasing resources in the development of peptide-based therapeutics, glimpsing the extraordinary potential of peptide molecules as a modern platform for drug development [24]. The availability of consolidated approaches for peptide drug discovery, easy-to-practice and cheap chemical methodologies for peptide synthesis and highly reproducible protocols for purifying peptides at a pharmaceutical grade, strongly boosted the interest of the pharma industry in approaching such a class of molecules as an attractive alternative to the conventional drugs, i.e., small organic molecules and large proteins (mainly antibodies) [25,26,27,28,29,30]. Peptides are fast succeeding as a second generation of pharmaceutics in biomedicine as they overcome the limitations of both small organic molecules and antibodies and combine their favorable properties. Compared to small organic molecules, which very often are responsible for establishing off-target molecular interactions, peptides display a significantly higher selectivity for their biological targets. Consequently, peptide drugs are usually safe and do not cause the serious undesired side effects often observed with small organic molecule-based pharmacological therapies. This issue is for instance faced with the use of VEGFR TKIs that very often are multitarget drugs exerting an inhibitory effect not only on VEGFR1/2 but also on other TKRs, consequently causing severe collateral effects [25]. In addition, compared to small organic molecules, peptides feature a more predictable metabolism, usually decomposing in nontoxic catabolites and therefore exhibiting a favorable toxicological profile [31]. In comparison to large protein therapeutics, peptide drugs share the high selectivity of monoclonal antibodies but, conveniently, are 100 times smaller in size, a key feature that results in a better distribution through the body, improved tissue penetration and better renal clearance, thus overcoming the major pharmacokinetic limitations of antibodies. In addition, peptides are usually poorly immunogenic or non-immunogenic [32]. The immunogenicity of anti-VEGF antibodies is a great concern, especially in long-term therapies applied to the treatment of ocular diseases [33]. The smaller size of peptide molecules is also convenient from a chemical point of view, as such a feature ensures ease of manipulation and a high level of control over chemical functionalization. Due to their small size, the formation of side products during chemical modification/functionalization of a peptide is limited, allowing high batch-to-batch reproducibility and homogeneity to be obtained compared to large protein-based therapeutics. The possibility to selectively modify peptide chemical structure is also a relevant advantage for the improvement of the pharmacological properties of peptides, switching from peptides to peptidomimetics [31]. Indeed, although being extremely promising as biomedical tools of the future for both diagnostic and therapeutic applications, peptide molecules have some drawbacks, especially related to their poor resistance to proteolytic degradation and poor cell membrane permeability, resulting in short half-lives and poor oral bioavailability. A lot of research in peptide chemistry has been devoted to the development of chemical strategies useful to obtain peptidomimetics with improved pharmacological properties, enhancing peptide resistance to proteases and cell membrane permeability [25,31]. For instance, shielding of a peptide molecule by conjugation to polymers or lipids, the introduction of unnatural amino acids (i.e., D-amino acids or C-α/N-α methylated amino acids), the installation in the backbone of peptide-bond surrogates (i.e., semicarbazides, 1,4-disubstituted 1,2,3-triazole, four-membered oxetane ring, thioamide or ester bonds), intramolecular cyclization and the insertion of secondary structure constraints (helix stapling or β-hairpin strands covalent bridging) are the traditional tools successfully exploited to enhance peptide stability and efficacy and successfully applied also to VEGF/VEGFR-targeting peptides [34,35]. Most recent advances in peptide drug therapy were devoted to improving protease stability and oral bioavailability, which is the most desirable route of drug administration [36,37,38]. In this context, co-delivery of permeation enhancers, inhibitors of gut enzymes, mucus- or cell-penetrating peptides, and the use of nano- and microscale delivery platforms were recently exploited as cutting-edge approaches to improve protease resistance and oral bioavailability of peptides [37,39,40]. The availability of a wide portfolio of selective chemistries allowing for the controlled modification of peptides has also paved the way for the exploitation of peptides as targeting systems in precision medicine applications, especially in oncology [41,42]. Peptide binders of tumor antigens, notably peptide binders of the VEGFRs which are overexpressed on cancer cells, could be exploited as homing units to selectively carry a cytotoxic drug or an imaging probe (or even both) to the tumor site [32]. Peptide–drug conjugates are cheaper and are more easily prepared and site-specifically modified/functionalized in comparison to antibody-based conjugates. Notably, the fine ability to chemically manipulate peptides in a highly controlled way allows the preparation of multi-modified peptides, suggesting the most innovative applications in dual-imaging and theranostics [43]. Peptide–drug conjugates exploiting peptides targeting VEGFRs as homing units have been described in the literature [44,45,46].

In the following paragraphs, the peptide design of molecules targeting VEGF or VEGFRs is reported. The described molecules are summarized in Table 1, which also reports the peptide name (or the number indicated in the original work), the amino acid sequence, the target, the mimicking region, the affinity (binding constant or IC_50_, only if a titration was performed against the target) and the reference to the original work.

## 4. Peptides Targeting VEGF

**Figure 2:**
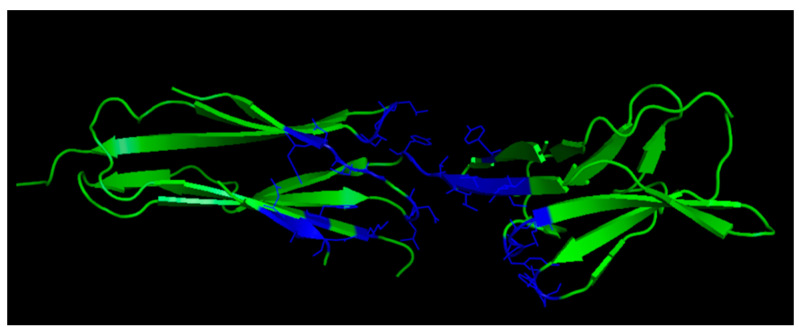
VEGF-A binding epitopes of VEGFR-2 (3V2A.pdb) [74]. VEGFR-2 domains 2 and 3 are reported in green; VEGFR-2 residues interacting with VEGF-A are highlighted in blue.

Several peptides targeting VEGF have been reported [73], but the structure-based design of VEGF peptide binders mimicking the VEGFR binding interface has been scarcely described so far, probably reflecting a difficult surface to be addressed by rational design methods. The VEGF-A binding epitopes of VEGFR-2 involve residues spreading over Ig-like domains 2 and 3 (Figure 2); they are mainly distributed around the region connecting the two receptor domains.

Starting from the complex of VEGF/VEGFR-2, the receptor residues within 4 Angstrom distance from the ligand were identified and reported in the linear sequence NGIDFNRDKFSGL. This sequence was submitted to the AntiCP webserver and then to the peptide ranker server with the aim of deriving a bioactive anticancer peptide. A molecular docking analysis was performed to find the more stable peptide–VEGF complex. The anti-VEGF peptide NGIDFNRDKFLFL was the best performer. To improve the conformational stability of the linear peptide, the selected sequence was grafted into the loop of disulfide-rich cyclic peptides SFTI-1 and MCoTI-II [47]. However, no biological information on peptide activity has been reported so far.

Other approaches were based on the design of peptides mimicking the surface of the interaction of VEGF synthetic inhibitors. For example, Gellman’s group focused on the molecular mimicry of Z-VEGF, a VEGF binder based on the Z-domain or affibody scaffold. In particular, they focused on the use of β-amino acids to be inserted in non-binding position, to improve proteolytic stability of the molecule. Z-VEGF interacts with VEGF with residues disposed on helices 1 and 2, so a helix–loop–helix α/β peptide was designed keeping the interacting residues and inserting a disulfide bond, a combination of aminoisobutyric residues, β^3^-residues and cyclic β-residues to improve the stability of the structural motif. A 39-mer peptide was obtained with a high binding affinity (0.11 μM) and was able to reduce VEGF-stimulated EC proliferation [48,75]. Other VEGF inhibitors such as a VEGF-binder nanobody [49] and the phage-derived peptide v114 [50] have been exploited for molecular mimicry. In this last case, the use of C^α^-tetrasubstituted α-amino acids was explored to derive a peptide with lower flexibility but improved proteolytic stability.

## 5. Peptides Targeting VEGF Receptors

**Figure 3:**
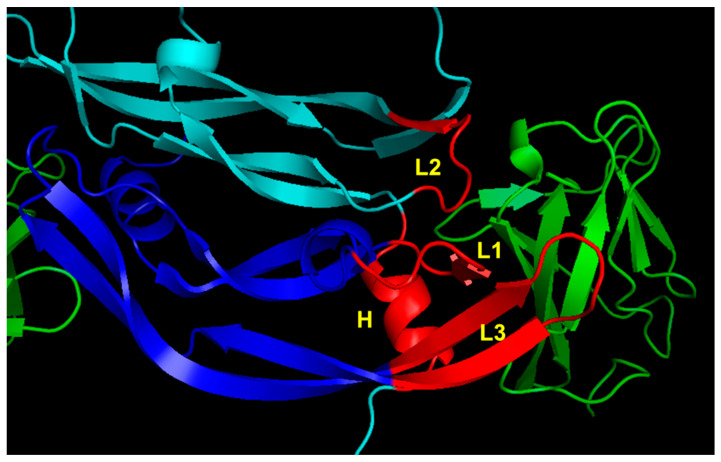
VEGFR-1 binding region of VEGF-A (1FLT.pdb) [76]. VEGF monomers are colored in cyan and blue. The VEGFR1D2 is reported in green. The VEGF-A regions involved in receptor recognition are highlighted in red and are the N-terminal α1 helix (H), loop 1 (L1), loop 2 (L2) and loop 3 (L3).

At first, the design of peptides targeting VEGF receptors was based on the X-ray structure of the complex between VEGF-A and domain 2 of VEGFR-1 (VEGFR1D2) [76]. The natural ligand interacts with the receptor with a discontinuous binding interface mainly involving four regions spread over the two monomers: the N-terminal α1 helix (residues: 17–25) and the loop involving residues 61–68 (loop 2) in one monomer, and strands β5 and β6 with the connecting loop (residues: 79–93; loop 3) and a short β-strand involving residues 43 to 48 (loop 1) of the other monomer (Figure 3).

**Figure 4:**
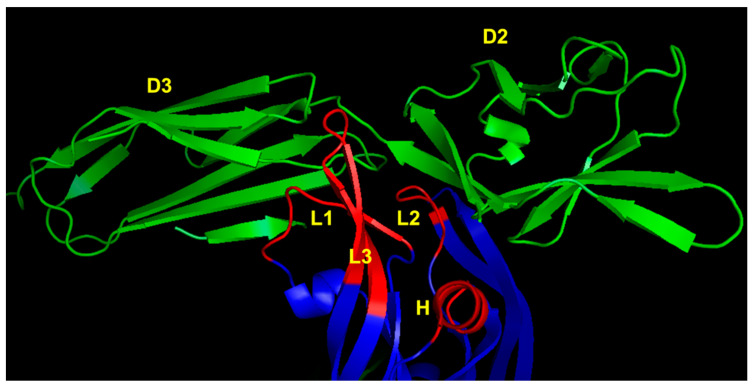
VEGFR-2 binding region of VEGF-A (3V2A.pdb) [74]. VEGF-A is represented in blue; VEGFR-2D23 is reported in green. The VEGF-A regions involved in receptor recognition are highlighted in red and are the N-terminal α1 helix (indicated as H), loop 1 (L1), loop 2 (L2) and loop 3 (L3). D2 and D3 denote domains 2 and 3 of VEGFR-2, respectively.

Successively, the X-ray structure of VEGF-A complexed to VEGFR-2 domains 2 and 3 (VEGFR2D23) was reported [74], expanding our knowledge on the molecular recognition between ligand and receptor and opening the way to novel design opportunities. In particular, the presence of domain 3 highlighted the role of VEGF binding residues that were undisclosed based on the complex involving VEGF and VEGFR1D2, such as that of the loop 1 epitope (Figure 4).

### 5.1. Peptide Mimetics of α1 N-Terminal Helix Region

**Figure 5:**
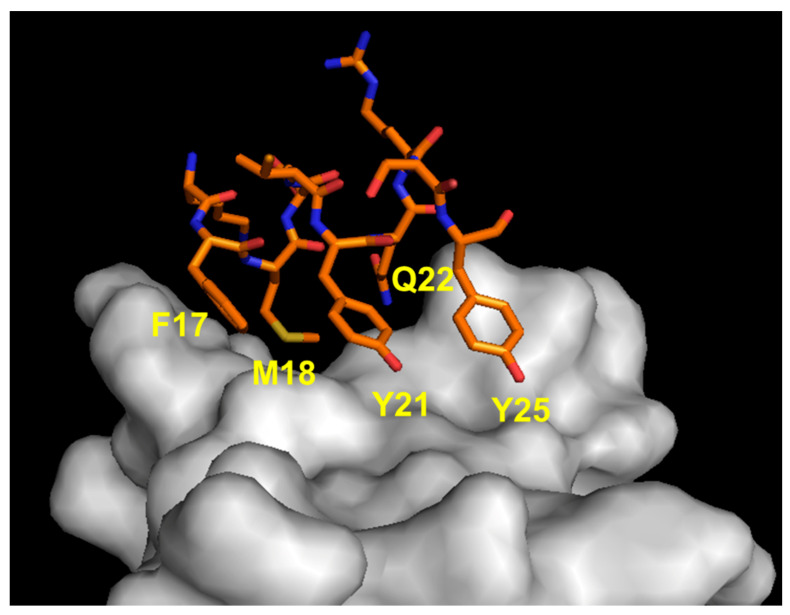
Detail of the interaction between VEGF α1 N-terminal helix region (orange) and VEGFR1D2 (gray). The VEGF-A-interacting residues are indicated (1FLT.pdb) [76].

We reported the structure-based design of peptides reproducing the VEGF-A N-terminal helix (Figure 5). The design strategy consisted in keeping the binding residues in the same spatial arrangements as in the natural complex, stabilizing the peptide helical conformation. The analysis of the VEGF-A/VEGFR1D2 complex revealed that N-terminal α1 residues Phe17, Met18, Tyr21, Gln22 and Tyr25 (VEGF numbering) are close to the receptor (<4.5 Å). These residues were selected as the binding residues. The peptide helical structure was stabilized by introducing N- and C-capping sequences, acetyl and amide groups at the N- and C-termini, respectively, and amino acids with high helical propensity at the non-binding positions. Then, we decided to replace Phe17 with Trp to increase the hydrophobic interactions with the receptor. A set of different peptides were synthesized, and peptides QK (Acetyl-KLTWQELYQLKYKGI-amide) [51] and MA (Acetyl-KLTWMELYQLAYKGI-amide) [52] were characterized in depth. Peptide QK is a proangiogenic molecule, and its biological and structural properties have been described in detail [77,78,79,80,81,82,83]. It is worth noting that an amino acid sequence with high homology with peptide QK but derived from a protein unrelated to VEGF presents a biological behavior similar to that of QK and is able to bind to VEGF receptors [84]. Peptide MA, instead, presents an antiangiogenic profile, being able to inhibit VEGFR-2 activity. It assumes a well-defined helical conformation in water, as assessed by circular dichroism (CD) and NMR characterization. It binds to VEGF receptors with an estimated binding affinity of about 46 μM, and an experimental model of the interaction with the receptor was derived by NMR analysis of the complex [85]. The peptide inhibits receptor signaling in ECs, proliferation and tumor growth in an experimental model of melanoma.

The observation that the three aromatic residues grafted on a stable helical scaffold could serve as a mimic of the α1 helical recognition motif suggested the development of a focused helical peptide library which provided 13-mer peptides able to antagonize the VEGF–VEGFR-1 interaction with an IC_50_ in the range 14–50 μM [53]. This study highlighted that the three aromatic residues could also be displayed on the helical scaffold in the arrangement (*i*, *i* + 4, *i* + 7) instead of (*i*, *i* + 4, *i* + 8), and that Trp to Tyr substitution improves the binding to the receptor.

A very interesting approach was pursued by Cai’s group using sulfono-γ-AA peptides. This class of peptidomimetics is based on a sulfono γ-substituted-N-acylated-N-aminoethyl amino acid unit and adopts a left-handed helix [86] which, opportunely decorated, has been demonstrated to inhibit several protein–protein interactions mediated by the α-helix motif [87]. In the case of the α1 helix of VEGF-A, the three aromatic residues (Trp/Phe, Tyr and Tyr) were inserted in the scaffold opportunely spaced out [54]. Three foldamers were designed, and two of them (designed as V2 and V3) were able to bind to VEGFR-1 and VEGFR-2, respectively, with high affinity (0.46 and 0.63 μM). Very interestingly, the two peptidomimetics showed opposite biological activity, V2 being a proangiogenic molecule and V3 being antiangiogenic, similarly to what has been observed with peptides QK and MA. Considering that these molecules are very resistant to proteolytic degradation, they appear to be good candidates for therapeutic application in vivo.

Peptides reproducing the α1 helix of VEGF-B were also reported. The native sequence (residues from 16 to 25) was used without amino acid modification but with two VEGF-B segments (residues 26–29 and 34–35) appended at the C-terminus, which should contain residues able to improve receptor binding affinity [55]. This 16-mer peptide (VGB1) presents a Cys just at the end of the helical segment which was linked through a disulfide bond to a Cys that was appositively added at the N-terminus. Circular dichroism analysis showed that the peptide is mainly disordered in water but the helical content increases in the presence of 30% TFE. A molecular modeling study of the complex between VGB1 and VEGFR-1 and VEGFR-2 was also reported [88]. Biological characterization in vitro and in vivo showed that the peptide VGB1 has antiangiogenic properties which are linked to its ability to bind to VEGFR-1 and VEGFR-2. This latter result should not be surprising, even considering that the natural ligand VEGF-B can only bind to VEGFR-1, as the N-terminal helix is an interaction region common to all ligands and receptor specificity is dictated from other ligand recognition sites.

Another example is represented by the peptide Vefin7, which was derived from VEGF-B sequence 17–25 by adding a lysine at the N-terminus and introducing V15A, I18V and R23A substitutions; then, a tetrameric form was prepared using a core of tetralysine. Vefin7 showed VEGF-B-mimetic properties. In fact, it binds with high affinity (K_D_ = 167 nM) to VEGFR-1 but not VEGFR-2; it is neurotrophic and neuroprotective and inhibits the proliferation of MCF-7 cells in vitro [56].

Peptides reproducing the α-helix fragments 13–25 of VEGF and 1–13 of Vammin were designed with the support of the AGADIR algorithm [57]. The interacting VEGF residues Phe17, Tyr21 and Tyr25 and the corresponding Vammin residues (Phe5, His9 and Ala13) were maintained, whereas the other amino acids were modified to induce the helical conformation. Cyclopeptide analogs were also designed by inserting a side-chain to side-chain lactam bridge introducing Glu and Lys residues in position *i*, *i* + 4 to stabilize the helical conformation. Peptide structures were mainly disordered in an aqueous solution and helical in 30% TFE, as determined by CD and NMR studies. Binding studies showed the ability of cyclic peptides to inhibit VEGF–VEGFR-1 interaction with an IC_50_ in the micromolar range.

The N-terminal helix of VEGF-C was also targeted to develop VEGFR inhibitors. Zanella and coworkers focused on this specific ligand as they noted that a specific electrostatic interaction between Asp123 (VEGF-C) and Arg164 (VEGFR-2) was present and that the natural sequence presented three residue pairs that could establish stabilizing interaction within the helix, suggesting the intrinsic stability of the natural sequence [58]. This sequence was modified according to a computational study mainly evaluating the binding free energy and then introducing α,α-dialkylated non-proteogenic amino acids (such as Aib) at the non-interacting position. Six derivatives were designed and tested for receptor binding and neo-vessel formation. Peptides Ac-Trp-(αMe)Asp-Asn-(αMe)Asp-Trp-Arg-Api-Thr-Trp-amide (Api = 4-aminopiperidine-4-carboxylic acid) and Ac-Api-Trp-Asp-Asn-(αMe)Asp-Trp-Arg-Api-Thr-Trp-amide were the most active.

### 5.2. Peptide Mimetics of Loop 1 Region

**Figure 6:**
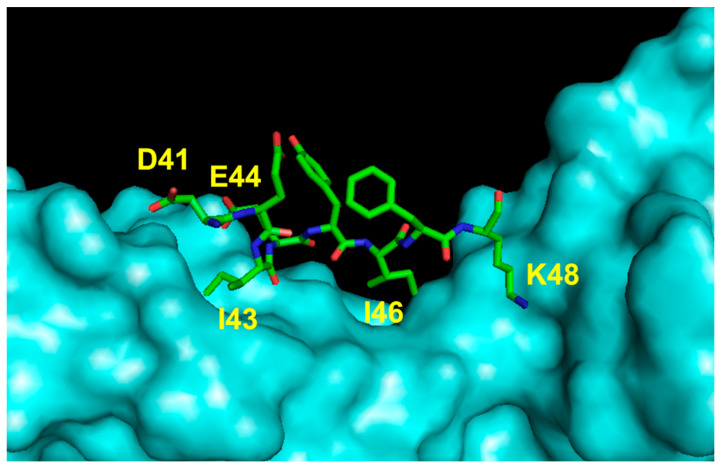
Detail of the interaction between VEGF loop 1 region and VEGFR2D23 (cyan). The VEGF-A-interacting residues are indicated (3V2A.pdb) [74].

The importance of the VEGF-A region encompassing residues 40 to 47 (loop 1) (Figure 6), which is mainly implicated in binding to domain 3 of the receptor, was pursued by Wang and coworkers in developing peptide inhibitors [59]. The linear native sequences of VEGF-A, VEGF-B and PlGF were constrained by introducing two cysteine residues at the peptide termini, showing elevated inhibition of the interaction between VEGF-A and VEGFR-1 with an IC_50_ ranging from 10 to 56 μM. The antiangiogenic effect of these peptides in comparison with the antibody bevacizumab was also tested in a functional assay using ECs (tube formation on matrigel). The peptide derived from the VEGF-B sequence performed as well as the antibody (50 μM vs. 6.5 μM), and its antiangiogenic activity was also determined in vitro and in vivo in an experimental model of human gastric cancer [89].

### 5.3. Peptide Mimetics of Loop 2 Region

**Figure 7:**
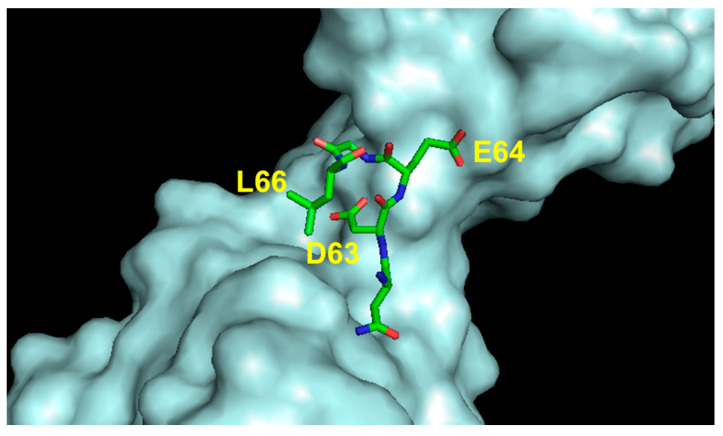
Detail of the interaction between VEGF loop 2 region and VEGFR2D23 (cyan). The VEGF-A-interacting residues are indicated (3V2A.pdb) [74].

The sequence 61–68 of VEGF-A (CNDEGLEC) adopts a loop structure (Figure 7) and is located close to the α1 helix. Structural and mutagenesis studies indicated that the residues Asp63, Glu64 and Glu67 are important for VEGF-A receptor recognition. In particular, Asp63 is involved in an electrostatic interaction with Arg224 of the VEGFR-1.

This region attracted the interest of Goncalves and coworkers [60], who tested linear and cyclic sequences. In particular, the cyclopeptide Ac-c[CNDEGLEC]-NH_2_ was found to be a weak binder for VEGFR-1, inducing the authors to optimize it by combining it with residues derived from the helix α1 (see infra).

### 5.4. Peptide Mimetics of Loop 3 Region

**Figure 8:**
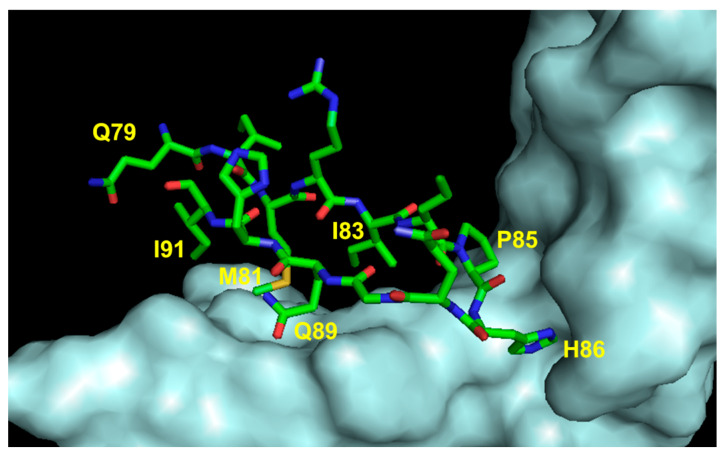
Detail of the interaction between VEGF loop 3 region and VEGFR2D23 (cyan). The VEGF-A-interacting residues are indicated (3V2A.pdb) [74].

Another VEGF-A region important for VEGFR recognition involves residues 79 to 93. In the natural ligand, this sequence assumes a hairpin structure involving strands β5 and β6 linked by a loop. The VEGF-interacting residues (Met81, Ile83, Lys84, Pro85, Gln89, and Gly92) have been identified (Figure 8).

Initially, a 17-mer cyclopeptide was designed based on the VEGF sequence 79–93 (cyclo-VEGI). In particular, the VEGF sequence 79–93 was head-to-tail cyclized, introducing the turn-inducing dipeptide D-Phe-Pro [61]. NMR characterization showed that the peptide is unordered in water but assumes a helical conformation in the presence of 30% TFE. However, cycloVEGI showed very interesting biological properties, being able to inhibit VEGF-dependent angiogenesis and glioma growth in vivo. A successive study showed that cyclo-VEGI inhibits bronchial artery remodeling in a mouse model of chronic asthma [90].

The approach of Garcia-Aranda and coworkers consisted in inducing hairpin stabilization in the natural VEGF sequence introducing an interstrand bridge at the peptide termini. Cyclopeptides were designed by replacing Arg82 and His90 with two cysteines, the pair Asp/Dap or two allylGly residues. Cyclopeptides presenting a disulfide or amide bond were obtained in the first two cases [62], whereas a hydrocarbon linker was obtained in the last case [63]. A conformational analysis was performed and showed that the peptides are flexible but have a tendency to adopt a β-turn structure. The cyclopeptides bind to VEGFR-1 with the amide-bridged analogs performing better than the heterodetic peptide.

Vicari and coworkers, in order to reproduce the conformation of the natural VEGF sequence, partially reversed the direction of the peptide backbone by inserting the sequence Ile80-Gly92 in the retro modality between the two terminal regions (Ile76-Glu79 and Glu93-Phe96, N- and C-terminus, respectively) so that Gly92, in the N-to-C backbone direction, follows Glu79 and Ile76 precedes Glu93 [64]. Two cysteines were inserted after Glu79 (and before Gly92) and before Glu93 (after Ile80) in order to constrain the peptide to the natural β-hairpin twist. A structural characterization was not performed, whereas the biological analysis revealed that the heterodetic cyclic peptide (VEGF-P3CYC) inhibits VEGFR-2 activity and blocks tumor growth in vivo. The peptide VEGFP3CYC and its retro-inverse analog showed antitumor and antiangiogenic effects in vitro and in vivo when used in combination with a peptide targeting the HER-2 receptor [91].

We addressed the design of peptides reproducing this region using the same approach applied to helical peptides QK/MA. In this case, we focused on the corresponding region of the VEGF homolog PlGF (residues: 87–100). The interacting residues were identified (Gln87, Leu89, Ile91, Pro97, Tyr99) and displayed on a β-hairpin peptide. The β-hairpin structure was stabilized by introducing an interstrand aromatic cluster (peptide HPLW: KQLLWIRSGDRPWYYTS) [65] or a disulfide bridge (peptide HPLC: KQCLWIRSGDRPWYCTS) [34]. In both cases, the natural loop sequence was inserted between the two strands to reproduce its natural conformation. The NMR structure of peptide HPLW free in solution and complexed to VEGFR1D2 was determined, revealing the correspondence with the design hypothesis [92,93]. In order to improve its metabolic stability, an analog presenting an interstrand triazole bridge, a tool that also improves β-hairpin stabilization [94,95,96], was prepared [35]. Functional characterization of peptide HPLC in ECs showed that it presents antiangiogenic activity, being able to inhibit VEGF intracellular pathways, VEGF prosurvival activity and cell proliferation [34]. Successively, several peptides reproducing the PlGF region 87–100 and presenting a mutation in position 94 (replacing a Gly with charged residues) were tested for their ability to bind VEGFR-1 [66]. The substitution of Gly94 with His improved receptor binding affinity by approximately 1 order of magnitude.

Mirassou and coworkers focused on Vammin, a VEGF isolated from snake venom, which selectively binds to VEGFR-2. The VEGF β-hairpin fragment corresponds to residues 69–80 in Vammin. The loop connecting the antiparallel β-strands presents one-residue insertion (Thr) with respect to VEGF, so this segment was classified as an antiparallel 4:6 β-hairpin showing a non-Gly β-bulge and overlapping β turns of types IV and I at the loop region [67]. The peptide design aims to reproduce the structure of the Vammin β-hairpin structure by incorporating stabilizing elements at non-binding positions. Charged residues Arg70 and Arg74 and the complete loop region (residues 72–77) were conserved, whereas as stabilizing elements, cross-strand disulfide bonds and Trp–Trp pairs were used at the non-hydrogen-bonded sites. Combining the stabilizing elements, four analogs were designed. The NMR characterization showed that the peptides assume a β-hairpin structure and the incorporation of both stabilizing elements induces higher β-hairpin populations.

## 6. Peptide Mimetics of Discontinuous Binding Surface

More sophisticated molecules have been designed with the aim of mimicking two or more binding regions of the discontinuous VEGF receptor interface on VEGF.

The loop 61–68 and the N-terminal α1 helix, in particular the aromatic residues Tyr21 and Tyr25, are in proximity. Goncalves and coworkers designed a series of cyclopeptides to mimic this specific region [60]. The cyclic scaffold mimics the loop 61–68 and keeps the important interacting residues Asp63/Glu64/Glu67. Furthermore, two aromatic residues were inserted in the non-interacting positions of the loop to mimic Tyr21/Tyr25 residues. The optimized cyclic peptide (c[YYDEGLEE]-NH_2_) displaces VEGF from the binding to VEGFR-1 with an IC_50_ of 40 μM, inhibiting intracellular receptor signaling and EC migration and capillary tube formation on matrigel. A structure–function relationship study was performed to identify residues important for activity, including NMR characterization of the complex peptide:VEGFR1D2 [97].

A peptide analog c[YKDEGLEE]-NHCH_2_CH_2_Ph(3,4-diOH) presenting an aromatic group at the C-terminus was recently characterized and showed an improved inhibitory activity compared to the parent peptide. The interaction mode with VEGFR1D2 was determined by NMR, and a dissociation constant was also estimated (K_D_ = 621 μM) [68]. The low affinity could be explained by considering that part of the binding residues should interact with VEGFR1D3 based on the structure of the complex between VEGF and the full extracellular domain of VEGFR-1 [98]. However, in a displacement test, an IC_50_ of 196 μM was determined [68].

A 25-mer bicyclic peptide was designed for the simultaneous reproduction of three VEGF regions: helix 16–26, loop 61–68 and the β-strand 102–107 [69]. The sequences of the three regions were connected by 6-aminohexanoic linkers. This choice implies that the loop sequence runs in a C-to-N direction, and for this reason, D-amino acids were used. Finally, the peptide was constrained by introducing one disulfide and Glu-Lys side-chain to side-chain bonds. The peptide interacts with VEGFR-1 with a measured IC_50_ of 52 μM (displacement test) and inhibits ERK1/2 activation and EC migration in vitro.

Peptides reproducing the discontinuous binding site of VEGF involving the α1 helix and the β-hairpin region 79–93 were also described. These two regions belong to different monomers in the natural ligand, but their N-termini are in close proximity (Val15 and Met78). The two native binding regions were covalently linked by spacers of diverse lengths. The biological characterization showed that these peptides showed a proangiogenic profile, and the most active compound, EP6, was characterized by NMR [70].

A peptide able to block VEGFR-1 and VEGFR-2 was designed in consideration of the VEGF binding interfaces involved in the molecular recognition of both receptors. In particular, loop 3 (residues 83–91) and residues 61 to 64 of loop 2 were considered important [71]. The peptide sequence is composed of the loop 3 residues flanked by two cysteines to induce loop stabilization through a disulfide bond constraint with the loop 2 residues appended to the C-terminus. The resulting heterodetic peptide (VGB: sequence CIKPHQGQHICNDE) was able to bind receptors expressed on both EC and carcinoma cell surfaces. In vitro and in vivo characterization showed that the peptide has an antiangiogenic biological activity with potential application as an anticancer agent. In fact, the peptide VGB strongly inhibited tumor growth in a murine model of carcinoma. Another peptide targeting both VEGFR-1 and VEGFR-2 was chemically designed by linking residues from loop 1 and loop 3 of VEGF-B, which are close in the space, with residues from the region 83–88 of VEGF-A. The hypothesis behind this design was that the fragment based on VEGF-B should allow for VEGFR-1 recognition, whereas the VEGF-A-derived fragment should allow for VEGFR-2 recognition. A 23-mer linear peptide consisting of the VEGF-A turn sequence flanked by the two VEGF-B segments at N- and C-terminal sides (loop and β-hairpin, respectively) was derived [72]. Receptor binding was demonstrated by flow cytometry using ECs and tumor cells. The antagonist activity was demonstrated by analyzing the inhibition of the receptor signaling in ECs and tumor growth in a murine model of carcinoma.

## 7. Conclusions

The VEGF/VEGFR molecular system has attracted great interest in drug discovery over the last few decades, and several molecules able to interact with either partner have been described, mainly antibodies, small organic molecules and, more recently, peptides. Here, we report a comprehensive overview of the peptide molecules targeting VEGF/VEGFRs developed so far by structure-based design approaches and the strategies pursued to develop them. The design and characterization of peptides mimicking the binding interface of the VEGF/VEGFR molecular complex allowed the accomplishment of structural and biological studies that deepened our understanding of the key elements responsible for the VEGF/VEGFR molecular recognition. Noteworthily, we expect that the design of even more sophisticated peptides targeting VEGF/VEGFRs will produce valuable drug candidates expected to enter clinical trials in the near future, providing novel options for biomedical applications both in the therapeutic and the diagnostic field.

## Figures and Tables

**Table 1:** Structure-based designed peptides targeting VEGF/VEGFRs.

Peptide	Sequence ^a^	Target	Mimicking ^m^	Affinity	Ref.
-	NGIDFNRDKFLFL	VEGF	VEGFR-2	-	[47]
α/β-VEGF-1	V(β^3^-Val)NK(β^3^-Phe)NKE(ACPC)**C**N (APC)RAIE(Aib)ALDPNL NDQQFH (Aib)KIW(APC)II(APC)D**C** ^b^	VEGF	Z-VEGF (VEGF affibody)	K_i_ = 0.11 μM	[48]
-	YY(Abu)AARAWSPYSSTVDAGDFRYWGQ-amide ^c^	VEGF	Nd42 (anti-VEGF nanobody)	K_A_ = 51 × 10^9^ M	[49]
Aib2	V(Aib)PN**C**DIHV(nL)WEWE**C**FERL ^d^	VEGF	V114* peptide	K_D_ = 4 nM	[50]
Kv114*	K(Aib)KK**C**DIHV(nL)WEWE**C**FERL ^d^	VEGF	V114* peptide	K_D_ = 540 nM	[50]
QK	Ac-KLTWQELYQLKYKGI-amide	VEGFR1/R2	α1 VEGF-A	K_D_ = 64 μM	[51]
MA	Ac-KLTWMELYQLAYKGI-amide	VEGFR1/R2	α1 VEGF-A	K_D_ = 46 μM	[52]
#29	Ac-SSEEFARNWAAIN-amide	VEGFR1	α1 VEGF-A	IC_50_ = 0.05 μM	[53]
V2	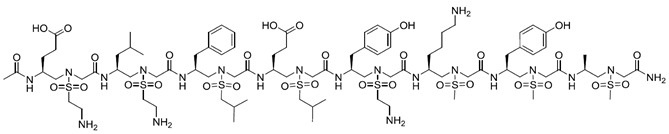	VEGFR1/R2	α1 VEGF-A	K_D_ = 0.46 μM	[54]
V3	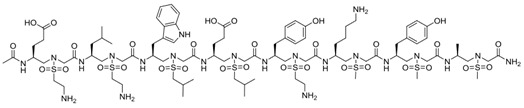	VEGFR1/R2	α1 VEGF-A	K_D_ = 0.63 μM	[54]
VGB1	**C**SWIDVYTRAT**C**QPRPL	VEGFR1/R2	α1 VEGF-B	-	[55]
Vefin7	KAVSWVDVYTAAT-scaffold ^e^	VEGFR1	α1 VEGF-B	K_D_ = 0.167 μM	[56]
#16	Ac-EV-cycle(NHCO)^3,7^[EKFM(O_2_) K] VYQRSY-amide	VEGFR1	α1 Vammin	IC_50_ = 36 μM	[57]
#5	Ac-W(^αMe^Asp)N(^αMe^Asp)WR(Api)TW-amide ^f^	VEGFR1/R2	α1 VEGF-C	-	[58]
#6	Ac-(Api)WDN (^αMe^Asp)WR(Api)TW-amide ^f^	VEGFR1/R2	α1 VEGF-C	-	[58]
#14	**C**FQEYPDEIEYI**C**K-amide	VEGFR1	L1 VEGF-A	IC_50_ = 50.4 μM	[59]
#18	Ac-**C**TVELMGTVAKQLVP**C**-amide	VEGFR1	L1 VEGF-B	IC_50_ = 10.4 μM	[59]
#19	**C**SEYPSEVEHM**C**S-amide	VEGFR1	L1 PlGF	IC_50_ = 56.0 μM	[59]
#2	Ac-**C**NDEGLE**C**-amide	VEGFR-1	L2 VEGF-A	-	[60]
Cyclo-VEGI	Cycle(fPQIMRIKPHQGQHIGE)	VEGFR1/R2	L3 VEGF-A	IC_50_ = 0.7 μM	[61]
#7	Ac-M-cycle(CH_2_NHCOCH_2_)^2,10^ [GIKPHQGQG]I-amide	VEGFR1	L3 VEGF-A	IC_50_ = 93.2 μM	[62]
#8	Acetyl-M(aGly)GIKPHQQ(aGly)I-amide ^g^	VEGFR1	L3 VEGF-A	IC_50_ = 42.3 μM	[63]
VEGF-P3CYC	Acetyl-ITMQ**C**GIHQGQHPKIRMI**C** EMSF-amide	VEGFR2	L3 VEGF-A	K_D_ = 11 nM	[64]
HPLW	KQLLWIRSGDRPWYYTS	VEGFR1/R2	L3 PlGF	K_D_ = 32 μM	[65]
HPLC	KQ**C**LWIRSGDRPWY**C**TS	VEGFR1/R2	L3 PlGF	-	[34]
HPLW2	Ac-KQLL(Bpg)IRSGDRP(OrnN3) YWTS-amide ^h^	VEGFR1/R2	L3 PlGF	-	[35]
PLGF2	QLLKIRSHDRPSYVE-amide	VEGFR1	L3 PlGF	K_D_ = 86 μM	[66]
C1C12W3W10	**C**RWNPRTQSWK**C**	VEGFR2	L3 Vammin	-	[67]
#7	c[YYDEGLEE]-amide	VEGFR1	L2/α1 VEGF-A	IC_50_ = 39.9 μM	[60]
#3	c[YKDEGLEE]-NHCH_2_CH_2_Ph(3,4-diOH)	VEGFR1	L2/α1 VEGF-A	IC_50_ = 196 μM	[68]
VG3F	KFMDVYQRSY(Ahx)elGedn**c**s(Ahx)E**C**RPK-amide ^i^	VEGFR1	α1/L2/L3 VEGF-A	IC_50_ = 52 μM	[69]
EP6	Ac-E(C-7Ahp-QIMRIKPHQGQHIGET S)KFMDVYQLKYKGI-amide ^l^	VEGFR1/R2	α1/L3 VEGF-A	K_D_ = 139 μM	[70]
VGB	**C**IKPHQGQHI**C**NDE	VEGFR1/R2	L2/L3 VEGF-A	-	[71]
VGB4	KQLVIKPHGQILMIRYPSSQLEM	VEGFR1/R2	L1/L2 VEGF-B + L3 VEGF-A	-	[72]

^a^ Residues in bold form a disulfide bond. ^b^ ACPC = 2-aminocyclopentanecarboxylic acid; Aib = 2-aminoisobutyric acid; APC = 4-aminopyrrolidine-3-carboxylic acid. ^c^ Abu = aminobutyric acid. ^d^ Aib = 2-aminoisobutyric acid; nL = nor-leucine. ^e^ scaffold = four sequences on tetraLys scaffold. ^f^ Api = 4-aminopiperidine-4-carboxylic acid; ^αMe^Asp = α-methyl-aspartic acid. ^g^ aGly = allylGlycine. ^h^ Bpg = bishomopropargylglycine; OrnN3 = L-azido-d-ornithine; Bpg and OrnN3 are linked through a 1,4 triazole ring. ^i^ Ahx = 6-aminohexanoic acid. ^l^ 7Ahp = 7- aminoheptanoic acid. ^m^ α1 = α1helix; L1 = loop 1; L2 = loop 2; L3 = loop 3.

## Data Availability

Data sharing not applicable.
